# Effects of L-carnitine on Polycystic Ovary Syndrome

**DOI:** 10.5935/1518-0557.20190033

**Published:** 2019

**Authors:** Saghar Salehpour, Leila Nazari, Sedighe Hoseini, Parya Bameni Moghaddam, Latif Gachkar

**Affiliations:** 1 Department of Obstetrics and Gynecology, Preventative Gynecology Research Center, Shahid Beheshti University of Medical Sciences, Tehran, Iran

**Keywords:** polycystic ovary syndrome, L-carnitine, hyperinsulinemia, HOMA index

## Abstract

**Objective:**

Polycystic ovary syndrome (PCOS) is a common disorder in women of reproductive age. This study investigated the effects of L-carnitine on the clinical and laboratory findings of women with PCOS.

**Methods:**

Eighty women diagnosed with PCOS between 2017 and 2018 by the Rotterdam Criteria were enrolled in the study; six were lost during the study. The participants were given L-carnitine 3 g daily (Pursinapharma, Iran) for three months. Blood samples were taken after overnight fasting at baseline and three months into the study to assess the levels of fasting glucose, insulin, triglycerides, high-density lipoprotein (HDL), low-density lipoprotein (LDL), free testosterone, dehydroepiandrosterone (DHEA), and the insulin resistance index (HOMA-IR). The patients were weighed before and after treatment and had their body mass index (BMI) calculated. Menstrual cycles and manifestations of hirsutism were also assessed.

**Results:**

The data showed a significant improvement in insulin sensitivity and decreases in serum LDL levels and the BMI after three months of treatment. There was a significant increase in serum HDL levels. More regular menstrual cycles and decreased hirsutism were also observed.

**Conclusion:**

It appears that treatment with L-carnitine might decrease the risk of cardiovascular events by normalizing metabolic profiles and the BMI.

## INTRODUCTION

Polycystic ovary syndrome (PCOS) is a common disorder that affects 15-20% of women of reproductive age. The characterization of PCOS may require ultrasound examination and the observation of other signs such as irregular menstrual cycles, hyperandrogenism leading to acne, alopecia, hirsutism, insulin resistance, dyslipidemias, android obesity, early pregnancy loss, and infertility (Raja-Khan *et al*., 2011; Stener-Victorin *et al*., 2013; Marshall & Dunaif, 2012).

Despite the relevance and prevalence of PCOS, there is no consensus on how the condition should be treated and managed. Different results have been reported from treatment protocols using metformin and statins. Treatments with myo-inositol and N-acetylcysteine have been recently described. Several methods have been used to treat women with PCOS, but there is no agreement on which is the most effective. These points stress the need for more research and studies on the matter (Banaszewska *et al*., 2009; 2011; Kumar *et al*., 2016; Salehpour *et al*., 2012; Yang *et al*., 2016; Unfer *et al*., 2012; Aquino & Nori, 2014; Artini *et al*., 2013).

Conventional treatments for PCOS, which include management of symptoms and clinical signs, have little effect on long-term complications such as cardiovascular disease and hyperinsulinemia. In recent years, complementary therapies including lifestyle changes, yoga, acupuncture, aromatherapy, homeopathy, weight loss, medicinal herbs, and vitamins have been used (Genazzani *et al*., 2004; Aquino & Nori, 2014; Ratnakumari *et al*., 2018; Baillargeon & Nestler, 2006).

Several studies have described an association between PCOS and insulin resistance. Central obesity has been described in 30-40% of women with PCOS, while hyperinsulinemia affects more than 80% of them. Insulin resistance increases significantly with obesity, which may disturb ovulation and increase androgen levels. Some treatments of PCOS with metformin, pioglitazone, and troglitazone have described improved ovarian function by managing hyperinsulinemia and insulin resistance. Increased sensitivity to gonadotropins due to increased insulin sensitivity may lead to spontaneous ovulation and pregnancy. Furthermore, treatments that decrease insulin resistance - such as protocols with metformin - may increase the fertility rates of women with PCOS (Genazzani *et al*., 2004; 2007; Motta, 2012; Naderpoor *et al*., 2015; De Leo *et al*., 2003; Steiber *et al*., 2004; Suvarna *et al*., 2016; Ou *et al.,* 2017; Vanella *et al*., 2000; Pillich *et al*., 2005).

Carnitine is a quaternary amine synthesized in the body from amino acids lysine and methionine. In living cells, this chemical agent can transfer fatty acids from the cytosol to the mitochondria to produce energy from fatty acids. Carnitine is often used as a micronutrient and is divided into two types: L-carnitine (active form) and D-carnitine (inactive form). L-carnitine plays an important role in glucose metabolism and oxidative stress. L-carnitine can also stabilize the mitochondrial membranes and prevent cell apoptosis (Ringseis *et al*., 2012; Ismail *et al*., 2014; Fenkci *et al*., 2008). Some authors have looked into the role of carnitine in the treatment of insulin resistance and in the accumulation of acetyl coenzyme A. Insulin resistance has been linked to the occurrence of carnitine deficiency during chronic metabolic stress conditions such as diabetes mellitus type 2 and obesity. Recent studies have reported decreased levels of L-carnitine in patients with PCOS and apparently significant correlations between lower levels of L-carnitine and greater chances of individuals with PCOS developing hyperinsulinemia (Bacurau *et al*., 2003; Wächter *et al*., 2002; Karlic & Lohninger, 2004; Jamilian *et al*., 2017).

This study aimed to assess the effects of L-carnitine on the clinical and laboratory parameters and metabolic profiles of individuals with PCOS.

## MATERIALS AND METHODS

Eighty women of reproductive age diagnosed with PCOS based on the Rotterdam criteria (Rotterdam ESHRE/ASRM-Sponsored PCOS Consensus Workshop Group, 2004a,b) were enrolled in the study at the gynecology clinic of the Taleghani Hospital between 2017 and 2018. All participants gave written consent before joining the study. The Ethics Committee of the Shahid Beheshti University of Medical Sciences (SBMU) approved the study. Patients with adrenal deficiency or other endocrine conditions and individuals on hormone therapy in the six months prior to the study were excluded.

The women included in the study received L-carnitine 3 g daily (Pursinapharma, Iran) for three months. The patients were not instructed to follow a specific diet or introduce lifestyle changes. Blood samples were taken after overnight fasting at baseline and three months into the study to assess the levels of fasting glucose, insulin, triglycerides, high-density lipoprotein (HDL), low-density lipoprotein (LDL), free testosterone, dehydroepiandrosterone (DHEA), and the insulin resistance index (HOMA-IR). The patients were weighed before and after treatment and had their body mass index (BMI) calculated. The HOMA index was calculated as [baseline glucose] x [baseline insulin]/22.5. Menstrual cycles and manifestations of hirsutism were also assessed.

Sample size was determined after consideration for type 1 statistical error <5% and type 2 statistical error <20%. Results were shown as mean values plus or minus SD (Standard Deviation). Statistical analysis was performed using statistical software package SPSS 21.0 (SPSS Inc., Chicago, IL, USA). A *P* value of 0.05 was considered significant.

## RESULTS

Eighty patients with PCOS who met the inclusion criteria were enrolled in the study. Six participants were lost during the study.

Data are shown as mean values ± SD. Table 1 provides a summary of baseline characteristics and results after three months of treatment. There were significant decreases in the levels of fasting glucose, insulin, triglycerides, LDL, and in the BMI and HOMA index. There was a significant increase in the level of HDL. Regular menstrual cycles were reported by 48.6% and 61.1% of the patients before and after treatment, respectively. No relevant side effects were reported during and after the treatment.

**Table 1:** Patient characteristics and results

	Pre treatment	Post treatment	*p* value
FBS (mg/dl±SD)	92.90±11.83	87.12±9.41	<0.001*
Free testosterone (ng/ml±SD)	0.483±0.149	0.467±0.144	0.232
Insulin (mUI/l±SD)	23.60±4.29	17.31±5.15	<0.001*
DHEA (micromol/l±SD)	1.454±0.190	1.406±0.336	0.252
Ferriman–Gallwey score	6.62±2.17	6.26±2.52	0.062
LDL (mg/dl±SD)	169.47±32.7	152.47±32.19	<0.001*
HDL (mg/dl±SD)	35.62±5.39	39.11±5.86	<0.001*
Triglycerides (mg/dl±SD)	202.03±45.31	166.92±40.76	<0.001*
BMI (kg/m^2^±SD)	28.28±2.6	26.82±2.46	<0.001*
Menstrual regularity (%)	48.6	61.1	<0.001*
HOMA-IR	96.22±18.18	67.04±22	<0.001*

* Statistically significant difference FBS: Fasting blood sugar, DHEA: Dehydroepiandrosterone  LDL: Low-density lipoprotein, HDL: High-density lipoprotein, BMI: Body mass index *SD:*standard deviation

## DISCUSSION

PCOS is a common disorder in women of reproductive age closely tied to insulin resistance, a condition associated with obesity, metabolic syndrome, gestational diabetes, type 2 diabetes, and cardiovascular disease. The management of PCOS may be challenging on account of the comorbidities associated with the disease. Recent studies have focused on the long-term complications of PCOS. Hormonal contraceptives, insulin-sensitizing drugs such as metformin, thiazolidinediones, myo-inositol, statins, orlistat, and N-acetylcysteine have been prescribed to women with PCOS, but their usage is limited due to side effects.

L-carnitine supplementation has been recently used in obese patients to enhance the metabolic cascade. In the body, L-carnitine is produced in the liver and kidneys and stored in the musculoskeletal system, heart, brain, and sperm. L-carnitine supplementation is used to increase energy consumption and reduce lipids and weight (Vanella *et al*., 2000; Pillich *et al*., 2005; Ringseis *et al*., 2012; Ismail *et al*., 2014; Fenkci *et al.,* 2008). L-carnitine plays an important role in glucose metabolism and oxidative stress. According to the literature, low serum levels of L-carnitine, even in non-obese women, may be associated with insulin resistance and hyperandrogenism (Salehpour *et al*., 2016; Celik*et al*., 2017; Samimi *et al*., 2016; Ismail *et al*., 2014).

This study looked into the effects of L-carnitine on the clinical and laboratory parameters of women with PCOS. The results showed a significant reduction in the BMI and serum levels of TG, LDL, FBS, and insulin, in addition to increased serum HDL levels. Improvement in menstrual cycle regularity was reported without drug-related side effects. According to the results, it appears that treatment with L-carnitine may have improved the hormonal and metabolic parameters of women with PCOS.

Ismail *et al*. (2014) reported that prescribing L-carnitine to clomiphene-resistant patients with PCOS improved the quality of ovulation, pregnancy rates, lipid profiles, and the BMI. It has been established that L-carnitine is safe and may be used to eliminate the long-term complications of PCOS. The long-term effects of L-carnitine for women with PCOS should be further evaluated. Studies with longer treatment cycles should be conducted to confirm the value of this therapy for women with PCOS at risk of metabolic syndrome and cardiovascular disorders.

## CONCLUSION

It appears that treatment with L-carnitine may decrease the risk of cardiovascular events by normalizing metabolic profiles and the BMI.

Acronyms:

1. FBS: Fasting blood sugar
2. BMI: Body mass index
3. HDL: High density lipoprotein
4. LDL: Low density lipoprotein
5. TG: Triglycerides
6. DHEA: Dehydroepiandrosterone
7. PCOS: Polycystic ovary syndrome
8. HOMA index: calculated as [baseline glucose] x [baseline insulin]/22.5.
9. SD: Standard Deviation
